# New Jersey State Veterinary Society

**Published:** 1890-04

**Authors:** A. T. Sellers

**Affiliations:** Secretary


*New Jersey State Veterinary Society.*—The regular semi-annual meeting of the New Jersey State Veterinary Society was held at the American House, Trenton. Meeting called to order by the President, Dr. E. Loblein. On roll-call, the following members answered to their names : Drs. Autenreith, Corlies, Krowl, Kuehne, Lowe, Loblein, Pacock, Sattler, McIntosh, Sellers, and Hopkins. The minutes of the last annual and special meeting, called by the President, were read and so adopted. The Committee on Jurisprudence reported that they held a consultation with Hon. R. Wayne Parker in reference to prosecuting illegally practising veterinarians in New Jersey. After considerable discussion it was moved and seconded that the following resolution be adopted :

*Resolved*, That this Society shall cause to be published in a newspaper in each county in this State a list of the veterinary surgeons who have registered in each county clerk's office, as provided for by Chapter XXIV, Laws of New Jersey, 1889, giving date and college from which they graduated, also a list of the "Existing Practitioners" who have registered in accordance with the law.

*Resolved*, That under existing circumstances, and by advice of counsel, Hon. Wayne Parker, it is deemed unwise at present to prosecute persons failing to comply with the provisions of Chapter XXIV, Laws of New Jersey, 1889.

The application for membership of Drs. Otto Von Lang and A. H. Downey were read and referred to the Board of Censors for investigation.

The resignation of Dr. Kuehne as secretary was offered, the Doctor stating that he intended to retire from practice and enter the drug business. It was moved and seconded that the resignation be accepted. Carried.

Nominations for Secretary for the balance of the term being in order, Dr. A. T. Sellers, of Camden, was nominated and duly elected.

Dr. Lowe moved, and was seconded, that the President appoint a committee to draw up resolutions in favor of the Army Veterinary Legislation Bill, now pending before Congress, and that the Secretary furnish copies to the New Jersey Representatives, also to Dr. R. S. Huidekoper, chairman of the Committee on Army Legislation of the United States Veterinary Medical Association. Carried.

The President appointed the Committee on Jurisprudence, and the following was presented :

RESOLUTIONS.

WHEREAS, The New Jersey State Veterinary Society, appreciating the monetary value of the live stock of the United States Army, and the importance of preventing the spread of contagious diseases, so disastrous to the efficiency of the army and to the live stock of citizens of this country ; and

WHEREAS, It is a well-known fact that glanders has been spread among the animals of citizens by the sale of army horses and mules, especially after the late war, and that during the war with Mexico glanders was conveyed to the horses of that country by the animals of our army ; and

WHEREAS, Many other diseases affecting the usefulness of horses, some of them preventable, or which would yield to suitable remedial measures, annually cause a heavy loss, and could be prevented by a proper system of sanitary and veterinary service ; and

WHEREAS, The position of Veterinary Surgeon in the United States Army is about equal to that of a non-commissioned officer, and is not in accordance with the dignity of a learned profession or the responsibility he should assume. Therefore, be it

*Resolved*, That the New Jersey State Veterinary Society, at a meeting held in Trenton, N. J., February 13th, 1890, heartily endorse the bill presented to Congress by the committee (Prof. R. S. Huidekoper, Chairman), of the United States Veterinary Medical Association, and we earnestly hope that our legislators in Congress will appreciate the necessity by enacting wise measures which will give to the army a perfect system of veterinary service and protection to the live stock of our citizens.

*Resolved*, That our Secretary shall send to our Representatives in Congress copies of the above resolutions.

The President appointed Drs. Hopkins and Voorhees as essayists for the next annual meeting.

Dr. Lowe read an able paper on "Heredity," which was discussed at some length by the different doctors.

It was decided to hold the next meeting at Newark.

On motion adjourned.

A. T. SELLERS, D.V.S., *Secretary.*

